# *PPI layouts*: BioJS components for the display of Protein-Protein Interactions

**DOI:** 10.12688/f1000research.3-50.v1

**Published:** 2014-02-13

**Authors:** Gustavo A. Salazar, Ayton Meintjes, Nicola Mulder

**Affiliations:** 1 Computational Biology Group, University of Cape Town, Cape Town, South Africa

## Abstract

**Summary:** We present two web-based components for the display of Protein-Protein Interaction networks using different self-organizing layout methods: force-directed and circular. These components conform to the BioJS standard and can be rendered in an HTML5-compliant browser without the need for third-party plugins. We provide examples of interaction networks and how the components can be used to visualize them, and refer to a more complex tool that uses these components.

**Availability:** http://github.com/biojs/biojs; http://dx.doi.org/10.5281/zenodo.7753

## Introduction

*“Proteins are the building blocks of life”*. This is probably the most commonly used analogy in the molecular biology world. Despite the danger of falling into a *cliché*, we will elaborate on this. A brick is not a building, not a room, not even a wall; in the same way, proteins rarely perform functions in isolation, it is their interactions which form the complex networks that are responsible for almost all cellular processes.

The number of reported Protein-Protein Interaction (PPI) networks has grown considerably, partly due to advances in high-throughput experimentation and partly due to the new predictions that result from these empirical data.

Various strategies have been used to store this data for ease of retrieval and analysis of PPI networks ^1^. For instance, IntAct ^2^ is an open source repository for molecular interactions reported in the literature or directly deposited by the user; the STRING database ^3^ includes physical and functional associations derived from genomic context, high-throughput experiments, coexpression analysis or the literature. Other PPI sources are described in: ^4– 9^.

The volume of data now contained in PPI repositories has made the analysis and understanding thereof a challenge that can be enriched by the use of visualization techniques. PPI networks have been found to follow a behaviour that in graph theory is referred to as *scale-free*, which uses a few highly connected nodes and many nodes with few interactors. This feature allows a user to predefine visualization strategies to highlight certain characteristics. This approach has been used for multiple tools, some are stand-alone applications (e.g. Cytoscape ^10^), Java applications that are web available via Applets (e.g.VisANT ^11^), or those developed on Flash (e.g. STITCH ^4^). Details about other tools, comparisons between them, highlighted features and supported layouts can be found in any of the following reviews: ^12– 14^.

The web technologies covered under the umbrella term of HTML5 allow the display of some of those layouts natively on the web in a fully interactive way. We have used these technologies to implement two of the most popular layouts for PPI network visualization: Force-directed and Circle.

The components were developed following the BioJS ^15^ standard and are freely available at its registry: http://goo.gl/064ChR, http://goo.gl/RGlRLB.

## BioJS components

We have implemented the standards defined by BioJS in the development of two components for the visualization of PPI networks. The use of BioJS gives visibility to the components so that they can be discovered by third parties interested in the visualization of protein networks on the web.

We consider the publication via the BioJS registry beneficial for both the potential user and the developer of the component. The user of the component will save time by adopting a component instead of developing it from scratch. The particular needs of the user may require the implementation of specifics which could contribute to the further development of the component in a communal effort. All this is in the nature of open source projects, but without visibility it would hardly ever happen.

Both PPI network visualizer components are implemented as wrappers of layouts included in the Data Driven Document (D3) library ^16^, a popular JavaScript library for the processing and visualization of data. Besides making these layouts follow the BioJS structure, most of the effort has been directed at providing methods that are closer to the PPI vocabulary (e.g. *addProtein()*, *addInteraction()*). Both components follow the same API (Application Program Interface) and therefore any script developed to display on one layout can be used on the other.

### Force-directed layout

The D3 library provides an implementation of the Barnes-Hut simulation in order to efficiently create a self-organized network. This algorithm performs better than others because it aggregates the forces of close nodes, to avoid individual calculation on the forces whenever possible. Its performance facilitates a smoother transition between the execution steps of the algorithm, creating an appealing visual effect of live self organization. We have used this generic network layout available in D3 to provide a set of methods and events to represent PPI networks.

**Figure 1.**
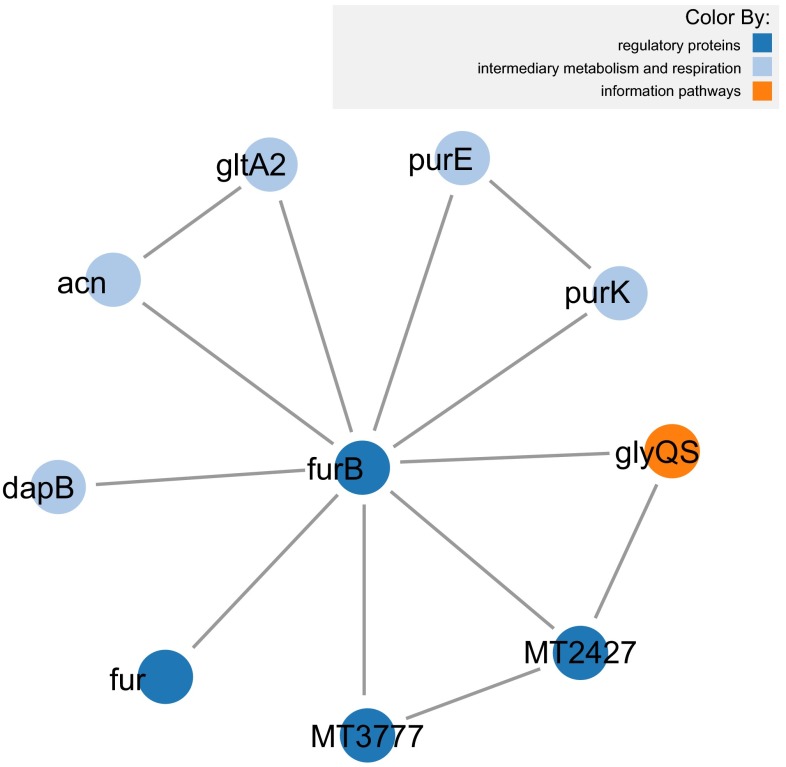
*Mycobacterium tuberculosis* proteins that interact with *furB* and the interactions between them (using force-directed layout).

Figure 1 displays a subset of proteins from the organism *Mycobacterium tuberculosis*, that interact with furB. It includes all the interactions between these proteins with each protein color coded by reported functional class. FurB is a zinc uptake regulation protein involved in repression of many genes involved in zinc homeostasis ^17^. FurB interacts with a number of different genes, with related functions, including the transcriptional regulator FurA, which represses transcription of katG, the catalase-peroxidase gene as well as itself, and the HTH-type transcriptional repressor SmtB, also involved in zinc homeostasis. FurB also interacts with the cAMP receptor protein, a global transcriptional regulator, although no link to metal ions has been annotated for this protein, a disruption in the protein results in slow growth. Additional interactions for FurB are with enzymes involved in amino acid biosynthesis (dapB, acn) or purine metabolism (purE, purK). One thing in common between these proteins appears to be their relation to growth.

BioJS provides instructions regarding the installation of each component including their required dependencies, style files and snippets of JavaScript code to be inserted into the web page where the component will be embedded. For example, installation instructions for the force-directed layout can be found on the *Installation* tab of the component page in the BioJS Registry ( http://goo.gl/064ChR).

The full script used to generate Figure 1 is available as a jsFiddle (a web resource for the online edition and display of snippets of JavaScript code) at this URL: http://jsfiddle.net/Bvh6k/1/. Below is a description of the main components of that code:

A developer should start by creating an instance of the component:



                        var instance = new Biojs.InteractionsD3({
   target: "example"
});
					


All the proteins in the graphic should then be added. The following example shows how to add the protein with UniProt id O05839 ( http://www.uniprot.org/uniprot/O05839). Note how the organism to which it belongs is included, along with which feature should be used for the label:



                        instance.addProtein({
      id: ʼO05839ʼ, organism: "MTB",
      gene_name:"furB",
      typeLegend:"gene_name"});
					


In the same way, the interactions should be added to the graphic, ensuring that the interactions are between proteins that have already been added. For instance:



                        instance.addInteraction(
      "O05839", "P0A582",
      {id: "P0A582_P0A582"}); 
					


Once all the proteins and interactions have been declared, the graphic can be restarted so it reflects the additions:



                        instance.restart ();
                    


The example also includes some instructions for coloring and format. For more details on those methods see the component on the BioJS registry: http://goo.gl/064ChR.

### Circle layout

**Figure 2.**
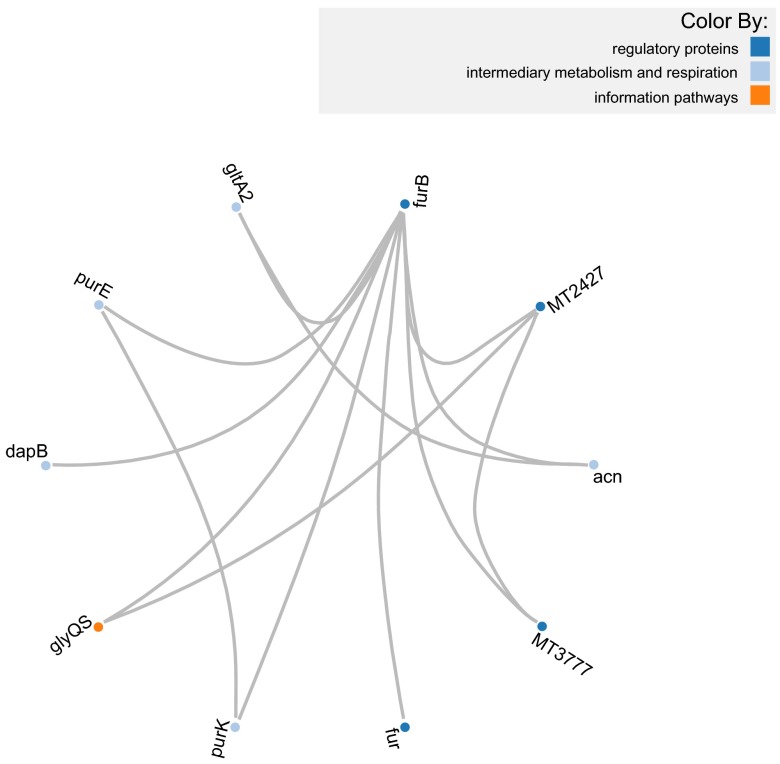
*Mycobacterium tuberculosis* proteins that interact with *furB* and the interactions between them (using Circle layout).

As mentioned previously, both components have the same API with the exception of methods that help to control the force layout of the first component which do not apply to the Circle layout. Thanks to this, the script to generate a PPI visualization of the same network is very similar in both. In order to demonstrate this we have created a second script that visualizes the same network as in Figure 1 but now on a Circle layout ( Figure 2). The only difference between both scripts is the declaration of the object. Instead of using the object *Biojs.InteractionsD3* it uses *Biojs.InteractionsBundleD3* and rest of the script is exactly the same. This can be seen in the fiddle: http://jsfiddle.net/J4CE7/1/.

## Use Case: PINV

We have used these two layouts and a few other BioJS components to create a web native application that allows the querying and visual exploration of PPI networks. It is called PINV: Protein Interactions Network Visualizer, and is freely available at http://biosual.cbio.uct.ac.za/pinv.html.

With PINV, we are looking to offer an alternative for the visualization of PPI data that takes advantage of the web to provide collaborative tools.

Currently PINV can display the PPI networks in three ways: the two layouts discussed above and through a table of the raw data. The addition of other layouts in the future is not complicated, thanks to the standards defined by BioJS and the adoption of the same API.

The same example used for Figure 1 and Figure 2 is available on PINV under this link: http://goo.gl/r4XpOS.

## Conclusions

One of the limitations of current web-based methods for PPI visualization is that they require third-party browser plugins. We demonstrate that the HTML5 standard provides enough functionality to render these networks in compliant browsers, without the need to install additional browser components. Adoption of the BioJS standard has the advantage of greater exposure to potential users, and an established set of features such as testing and documentation. Finally, the components abstract much of the details of rendering complex scalable vector graphics (SVG) behind an intuitive API, allowing users to focus on building richer applications.

## Software availability

Zenodo: BioJS components for the display of Protein-Protein Interactions, doi: 10.5281/zenodo.7753 ^18^.

GitHub: BioJs, http://github.com/biojs/biojs.
