# Supplementary material for: PPI layouts: BioJS components for the display of Protein-Protein Interactions
Source: F1000Res. 2014 Feb 13;3:50. [Version 1] doi: 10.12688/f1000research.3-50.v1 (PMC4103490; doi:10.12688/f1000research.3-50.v1)
Supplement: Peer review history

# Peer review: Referee response for version 1

**DOI:** 10.5256/f1000research.3677.r4104

**Published:** 2014-03-28

**Authors:** Hagen Blankenburg

**Affiliations:** 1 Center for Biomedicine, European Academy Bozen/Bolzano (EURAC), Bolzano, Italy

The two JavaScript components presented in this article facilitate the visualization of interaction networks in HTML5-enabled web browsers without requiring any third-party plugins like Flash. As they follow the BioJS standard, they are listed in the BioJS registry and should be interoperable with other components.

The manuscript is comprehensibly written and, despite the length limitations, successfully introduces both tools and their basic usage. The availability of the source code and particularly the jsFiddle live demo setup enable the interested reader to explore the software without complicated installation procedures.

In the following, I will list a few comments that might help to assure that the tools unlock their full potential. Note that while there is an overlap to the review by Laurent Gatto, I will repeat certain aspects for the sake of completeness.

**Interoperability with other BioJS components / PINV:**

The ability to show additional information about protein interactors (e.g., database identifiers, cross-references) and their interactions (e.g. links to source databases and publications, confidence scores) is what distinguishes a truly interactive network display from a nice visualization. In contrast to the two basic examples currently provided, PINV nicely demonstrates that this can be accomplished. Given that BioJS is essentially about modularity and re-usability of code, it would be great if the authors could provide more detailed examples that illustrate how their layout components can be combined with other BioJS components, e.g., to visualize additional information in popups. If manuscript space permits, additional commented source code files or a jsFiddle setup would be great and would further ease the application of the tools.

***M. tuberculosis example:***

As part of the BioJS collection, the manuscript generally seems to be written for computer scientists / developers rather than biologists. As such, the paragraph describing the *M. tuberculosis* interaction network example is too extensive and does not fit the focus of the remaining article. This example could be shortened and moved into the corresponding figure legends, making room for the aforementioned, more thorough description of other parts.

**Cytoscape.js:**

While the authors mention similar software like Cytoscape and VisANT, the HTML5 library Cytoscape.js ( http://cytoscape.github.io/cytoscape.js/) and its predecessor Cytoscape Web ( http://cytoscapeweb.cytoscape.org/) are omitted, although they are likely the closest competitors. 

**Source code examples:**

From the manuscript or the source code examples it is not obvious why the UniProtKB accession numbers and the organism information is provided, as neither is used or shown in the examples. It should be clarified, if tools require this information or if this might be used for connecting with other BioJS components.

**Title:**

Looking only at the title and the abstract, it appears that “PPI layouts” is the name of the BioJS component that is described in the article. As this is not the case and the name “PPI layouts” is never used again, it could be removed from the title to prevent confusion.

I have read this submission. I believe that I have an appropriate level of expertise to confirm that it is of an acceptable scientific standard.

# Peer review: Referee response for version 1

**DOI:** 10.5256/f1000research.3677.r3688

**Published:** 2014-03-10

**Authors:** Laurent Gatto

**Affiliations:** 1 Cambridge Centre for Proteomics, University of Cambridge, Cambridge, UK

The manuscript *PPI layouts:BioJS components for the display of Protein-Protein Interactions* by Salazar and colleagues briefly illustrate the BioJS component for the interactive html5-based visualisation of PPI.

The manuscript is easy to read and provides a quick, yet useful introduction to the BioJS PPI visualisation tools. The component described in the manuscript and the BioJS project in general are a welcome initiative. The PPI interaction tools was straightforward to assess thanks to support for the online edition, simple implementation, and its distribution to the bioinformatics community under an open access license.

Below, I have a few suggestions:

**BioJS component section**

In the first paragraph the sentence "The use of BioJS gives visibility to the components so that they can be [discovered] by third parties...". Do the authors mean that the individual component will benefit from the BioJS visibility, or is there a specific discovery mechanism?

***M. tuberculosis* example network**

While I appreciate that it is not easy to find the right balance between a simple yet biologically relevant illustration of the tool, I believe that the paragraph that describes the interaction network could be improved. For example, some proteins are named and described while others are not.

While reading the description of the code generating the network in Figure 1 and 2 and using the jsFiddle instance, several questions arose that could be addressed in the manuscript.

- the authors provide a UniProt identifier. Could the nodes be links to the relevant protein UniProt pages? Could other identifiers be used and how would the links to other arbitrary pages be defined?
- What is the use of the species name in the protein instance definition?  Is it mandatory? Are there examples of PPIs with multiple species?
- Graph annotation. Is it possible to use different symbols for the nodes? How to annotate the edges (through colours and/or labels)? Any comments on the use of node colours as a means to illustrate additional meta-data: are specific colour palettes (for continuous data for example) or transparency available?

**PINV**

The PINV application is a step further than the examples described in the manuscript, that possibly deserves a bit more description in the text and could guide the reader on how to build up from simple cases to more complex applications.

**Before Figure 2**

Instead of using the object Biojs.InterationsD3 it uses Biojs.InteractionsBundleD3 and [the] rest of the script is exactly the same.

**Compliant browsers**

The authors repeat the requirement for compliant browsers. I was wondering if examples of compliant browsers could be provided to guide users new to html5 and issues related to browser compatibility.

I have read this submission. I believe that I have an appropriate level of expertise to confirm that it is of an acceptable scientific standard.
